# Genipin in an Ex Vivo Corneal Model of Bacterial and Fungal Keratitis

**DOI:** 10.1167/tvst.10.9.31

**Published:** 2021-08-26

**Authors:** Elena Koudouna, Marcela Huertas-Bello, Cristian Nicolas Rodriguez, Sandra Consuelo Henao, Myriam Lucia Navarrete, Marcel Yecid Avila

**Affiliations:** 1 Department of Ophthalmology, Faculty of Medicine, Bogota DC, Universidad Nacional de Colombia, Bogota Colombia; 2 Structural Biophysics Group, School of Optometry and Vision Sciences, Cardiff University, Cardiff, Wales, UK; 3 Department of Microbiology, Faculty of Medicine, Bogota DC, Universidad Nacional de Colombia, Bogota Colombia

**Keywords:** Genipin, corneal crosslinking, corneal infectious keratitis, bacterial keratitis, fungal keratitis, *Staphylococcus aureus* corneal infection, *Pseudomonas aeruginosa* corneal infection, *Candida albicans* corneal infection, corneal ulcer

## Abstract

**Purpose:**

To determine whether genipin (a natural crosslinker) could reduce the colonization and proliferation of bacteria and fungi in an ex vivo model of corneal infection.

**Methods:**

This study, using an ex vivo model of bacterial and fungal keratitis, investigated the antimicrobial efficacy of genipin crosslinking. Excised corneoscleral buttons were wounded by scalpel incision and subsequently infected with *Staphylococcus aureus*, *Pseudomonas aeruginosa,* or *Candida albicans*. After inoculation, corneas were treated with genipin for 24 hours at 37°C. Histologic examinations were carried out, and the number of viable colony-forming units (CFU)/cornea was determined.

**Results:**

Genipin exerts bactericidal action against *S. aureus* and *P. aeruginosa*, as well as fungicidal action against *C. albicans* and significantly reduced the CFU compared to contralateral eyes that received saline treatment (*P* < 0.05).

**Conclusions:**

These data identify genipin as a novel ocular antimicrobial agent that has the potential to be incorporated into the therapeutic armamentarium against microbial keratitis.

**Translational Relevance:**

This study provided evidence for the antimicrobial and antifungal properties of genipin as an alternative crosslinker that could be used in the management of infectious keratitis.

## Introduction

Infectious keratitis, a corneal disease, is one of the leading causes of visual impairment and irreversible corneal blindness worldwide.^1^ Although the epidemiology of corneal infectious keratitis is complicated and varies according to geographical location, a conservative estimate of 1.5 to 2 million new cases annually in developing countries has been reported.^2^^,^^3^ In the United States, the financial burden of infectious keratitis is at least $175 million per year with approximately 1 million clinical visits annually^4^; this burden falls more heavily on developing countries.^5^^–^^8^ The major predisposing risk factors underlying the etiology and pathogenesis of microbial keratitis include contact lens wear, ocular trauma, ocular surface diseases, long-term use of immunosuppressive medications, and previous ocular surgery.^9^^–^^11^

A broad diversity of causative microorganisms, including bacteria, fungi, viruses, and protozoa have been implicated in infectious keratitis or corneal ulceration.^10^^–^^12^ Among the principal bacterial pathogens are *Staphylococcus aureus* (*S. aureus*), *Pseudomonas aeruginosa* (*P. aeruginosa*), and *Streptococcus pneumoniae*.^9^^–^^14^ *P. aeruginosa,* characterized as one of the most pathogenic ocular microbes, can cause corneal perforation in just 72 hours.^15^^,^^16^ Fungal agents such as *Fusarium, Aspergillus,* and *Candida* species are most commonly associated with mycotic keratitis.^11^^–^^13^ The yeast *Candida* is one of the most common fungi encountered in eye banks and among contact lens wearers.^14^^,^^15^ Thus infectious keratitis is considered a serious medical condition that, if not treated appropriately, can result in catastrophic complications including corneal scarring, eye perforation, endophthalmitis, and ultimately loss of the entire ocular globe and vision.^11^^,^^17^^,^^18^ Standard medical treatment involves the use of topical or systemic antibiotics, but the visual outcome is often poor.^19^^–^^21^ In developing countries, where prevalence is higher, access to specialized care is limited, and antimicrobial treatment is prohibitively unaffordable or unavailable.^3^^,^^5^^,^^6^ To make matters worse, the increasing emergence of multi-drug-resistant pathogens is another major challenge leading to higher rates of morbidity.^22^^–^^24^ In severe cases of medically uncontrollable infectious keratitis, surgical interventions and corneal keratoplasty are the last therapeutic alternatives; however, these are often associated with poor visual outcomes and increased risks of graft rejection/failure.^25^^–^^27^ Moreover, major shortage of corneal graft, with only one in 70 patients worldwide having access to donor tissue is an additional challenge.^28^

The poor prognosis for both medical and surgical treatments of corneal infectious keratitis urges the development of novel, unconventional therapeutic approaches to treat the condition and tackle drug resistance. The photoactivated chromophore crosslinking (PACK-CXL) approach, which uses riboflavin and UV-A light, has recently been proposed as an adjuvant therapy for resistant, nonresolving infectious keratitis.^29^^–^^34^ The combination of UV radiation and the crosslinking effect can synergistically kill microbes and protect the cornea against enzymatic degradation, preventing further tissue destruction.^35^^–^^38^ Nevertheless, in vitro and in vivo studies have demonstrated mixed results,^39^^–^^43^ and further studies are sorely needed to fully ascertain the therapeutic profile of PACK-CXL. Concerns include the potentially harmful effects of ultraviolet radiation and the risk of endothelial dysfunction, particularly in thin corneas,^44^^–^^47^ as well as the risk of herpes simplex viral keratitis reactivation after PACK-CXL.^48^^–^^50^ Recently, an alternative photodynamic therapy using Rose Bengal as the photosensitizer has also been proposed as an adjunctive treatment in cases with aggressive infectious keratitis.^51^^,^^52^ A limiting issue with the application of photodynamic therapy is the restricted tissue penetration and the strong dependence for efficacy on the distance between target and light source, which raises concerns about nonhomogeneous, incomplete microbial inactivation/death.^53^^,^^54^ Also important in relation to the concept of using photodynamic therapy are practical issues that currently limit its adoption in the developing world, where the burden of infectious keratitis is most severe, and ideally where a therapeutic approach is needed that not only is effective but also is affordable and can be easily applied.^3^^,^^5^^,^^6^

Genipin, a natural crosslinking agent, lately has been receiving great attention in biomedical applications on account of its stability, biocompatibility, and safety.^55^^–^^61^ Previously, we demonstrated effective crosslinking and stiffening of the cornea by genipin, ex vivo and in vivo, with minimal toxicity.^62^^–^^64^ Studies on sclera have also demonstrated successful stiffening and biocompatibility in rat,^65^ tree shew,^66^ pig,^67^^,^^68^ rabbit,^69^ and guinea pig tissue,^70^ supporting the prospect of sclera genipin crosslinking for the treatment of glaucoma and myopia. In addition, genipin exhibits several key pharmacological properties, including anti-inflammatory,^71^^–^^76^ antioxidant,^77^^,^^78^ antiangiogenic,^79^ and antimetastatic^80^^,^^81^ activities, highlighting its therapeutic value for the treatment of various diseases. Notably, genipin possesses antimicrobial properties, because genipin-crosslinked nanocomposite films restricted and inhibited *E. coli* and *L. monocytogenes* bacterial growth in fresh pork meats.^82^

Here we investigated the antimicrobial effects of genipin against *S. aureus*, *P. aeruginosa* and *Candida albicans (C. albicans)* in an ex vivo porcine corneal model of bacterial and fungal keratitis and discovered that it successfully inhibited the growth of all three pathogens. This highlights the unexploited potential benefits of genipin crosslinking for the treatment and management of severe microbial keratitis. Elucidating the bactericidal and fungicidal mode of action of genipin against these pathogens will certainly aid the development of an exciting, new therapeutic paradigm for the management of corneal keratitis, particularly in severe, nonresponsive cases.

## Materials and Methods

Ethical approvals for this study were granted by the Faculty of Medicine Ethical Committee, (approval acts: 004-039-19 and 019-212) and by the Faculty of Veterinary Medicine and Zootecnics ethical committee (approval act: CB-FMVZ-UN-004-2021), at the Universidad Nacional de Colombia, Bogota. This study was also conducted in accordance with the statement of the Association for Research in Vision and Ophthalmology for the use of animals in research.

### Bacterial and Fungal Strains

Clinical isolates of *S. aureus* (ATCC 25923), *P. aeruginosa* (ATCC 27853), and *C. albicans* (ATCC 90028) were obtained from the American Type Culture Collection (Labcare de Colombia Ltd, Cota, Colombia). The bacterial strains were cultured on brain-heart infusion (BHI) agar (Suministros Clinicos ISLA S.A.S, Bogota, Colombia) at 37°C overnight and subsequently maintained at 4°C. One day before infection of ex vivo corneas, one colony was subcultured into fresh BHI agar and incubated at 37°C overnight. On the day of corneal infection, a bacterial suspension of 0.5 McFarland, which corresponds to approximately 1.5 × 10^8^ colony-forming units per milliliter (CFU/mL) was prepared. *C. albicans* was cultured at 37°C in potato dextrose agar (Suministros Clinicos ISLA S.A.S) and then maintained at 4°C. Two days before use in experiments, one colony was subcultured into fresh potato dextrose agar and incubated at 37°C. An inoculum suspension of about 1 × 10^3^ CFU/mL was prepared in RPMI 1640 medium, buffered with 0.165 M MOPS [3-(N-morpholino) propanesulfonic acid] containing l-glutamine and lacking sodium bicarbonate (Gibco ThermoFisher Scientific, Bogota, Colombia).

### Genipin Susceptibility Testing – Evaluation of Antimicrobial Activity and Determination of the Minimum Bactericidal/Fungicidal Concentration

The antimicrobial activity of genipin against *S. aureus*, *P. aeruginosa,* and *C. albicans* was determined by using the broth dilution method as described previously and according to the Clinical and Laboratory Standards Institute guidelines for in vitro susceptibility testing of bacterial pathogens.^83^^–^^85^ In accordance with the guidelines, a wide range of high- and low- genipin concentrations was tested. For evaluation of the antibacterial activity of genipin, a stock solution was prepared by dissolving genipin (Challenge Bioproducts Co., Douliu City, Taiwan) in 30% dimethyl sulfoxide (DMSO; Sigma-Aldrich Corp., St. Louis, MO, USA) to a final concentration of 25 mg/mL. Thereafter, serial dilutions were made to obtain the following six concentrations: (a) 12.5 mg/mL in 15% DMSO, (b) 6.25 mg/mL in 7.5% DMSO, (c) 3.12 mg/mL in 3.75% DMSO, (d) 1.56 mg/mL in 1.87% DMSO, (e) 0.78 mg/mL in 0.94% DMSO, and (f) 0.39 mg/mL in 0.47% DMSO. A bacterial suspension of 1 × 10^5^ CFU/mL was prepared and inoculated into each well. Appropriate quality controls were also included for each strain: (1) the bacterial inoculum and Mueller-Hinton broth (nutrient media; Químirel Quimicos y Reactivos S.A.S, Bogota, Colombia); (2) the bacterial inoculum, nutrient media and 7.5% DMSO; (3) the bacterial inoculum, nutrient media and commercial antibiotics (for *S.aureus,* vancomycin at a concentration of 5 µg/mL and for *P. aeruginosa,* tobramycin (Tecnoquímicas S.A., Cali, Colombia) at a concentration of 3 mg/mL. The plates were carefully mixed and incubated at 37°C for 24 hours.

For the antifungal susceptibility testing, a genipin stock solution of 25 mg/mL dissolved in 15% DMSO was prepared, and thereafter serial dilutions were made as described above. A fungal suspension of 1 × 10^3^ CFU/mL was prepared and inoculated into each well. Appropriate quality controls were also included: (1) the fungal inoculum and nutrient media (RPMI 1640 buffered with 0.165 M MOPS containing l-glutamine and lacking sodium bicarbonate); (2) the fungal inoculum, nutrient media and 7.5% DMSO; (3) the fungal inoculum, nutrient media and amphotericin B at a concentration of 2.5 µg/mL. The plates were carefully mixed and incubated at 37°C for 48 hours.

The minimum bactericidal/fungicidal concentration (MBC/MFC) of genipin, which is defined as the lowest concentration that results in more than 99.9% microbial death, was determined by spreading broth aliquots from each well on Mueller-Hinton agar (Químirel Quimicos y Reactivos S.A.S, Bogota, Colombia) plates and incubated again at 37°C for 24 hours.

### Isolation and Preparation of Porcine Corneas

Heads of recently slaughtered pigs were obtained from a local abattoir (BLE Fridge, Ltd., Bogota, Colombia) and kept in a cooler box until arrival at the laboratory. The eyeballs were carefully dissected, kept in pairs and subjected to a decontamination protocol that involved an initial rinse of the eyeball with sterile saline solution (Baxter, Ltd., Marsa, Malta), followed by disinfection with 5% povidone iodine (Tecnoquimicas, Ltd, Cali, Colombia) for five minutes and a subsequent wash with sterile saline solution. Then, the globes were immersed in 0.046% hypochlorous acid (Neutroderm, Aquilabs S.A., Bogota, Colombia) for five minutes and washed with saline solution. Using a sterile surgical scalpel blade (Paramount Surgimed Ltd, Hannover, Germany), the cornea was wounded by creating three incisions vertically and three incisions horizontally, as previously described by Pinnock and colleagues^86^ in their ex vivo corneal model for microbial keratitis.^87^ The corneoscleral button was then carefully excised, placed into a sterile 35 mm dish, and drops of 5% povidone iodine solution were applied to both the epithelial and endothelial site for five minutes, followed by washing with sterile saline solution. After that, drops of 0.046% hypochlorous acid were also applied, and the corneal buttons were washed with saline solution.

### Infection of Ex Vivo Porcine Corneas and Genipin Treatment

**Figure 1.**
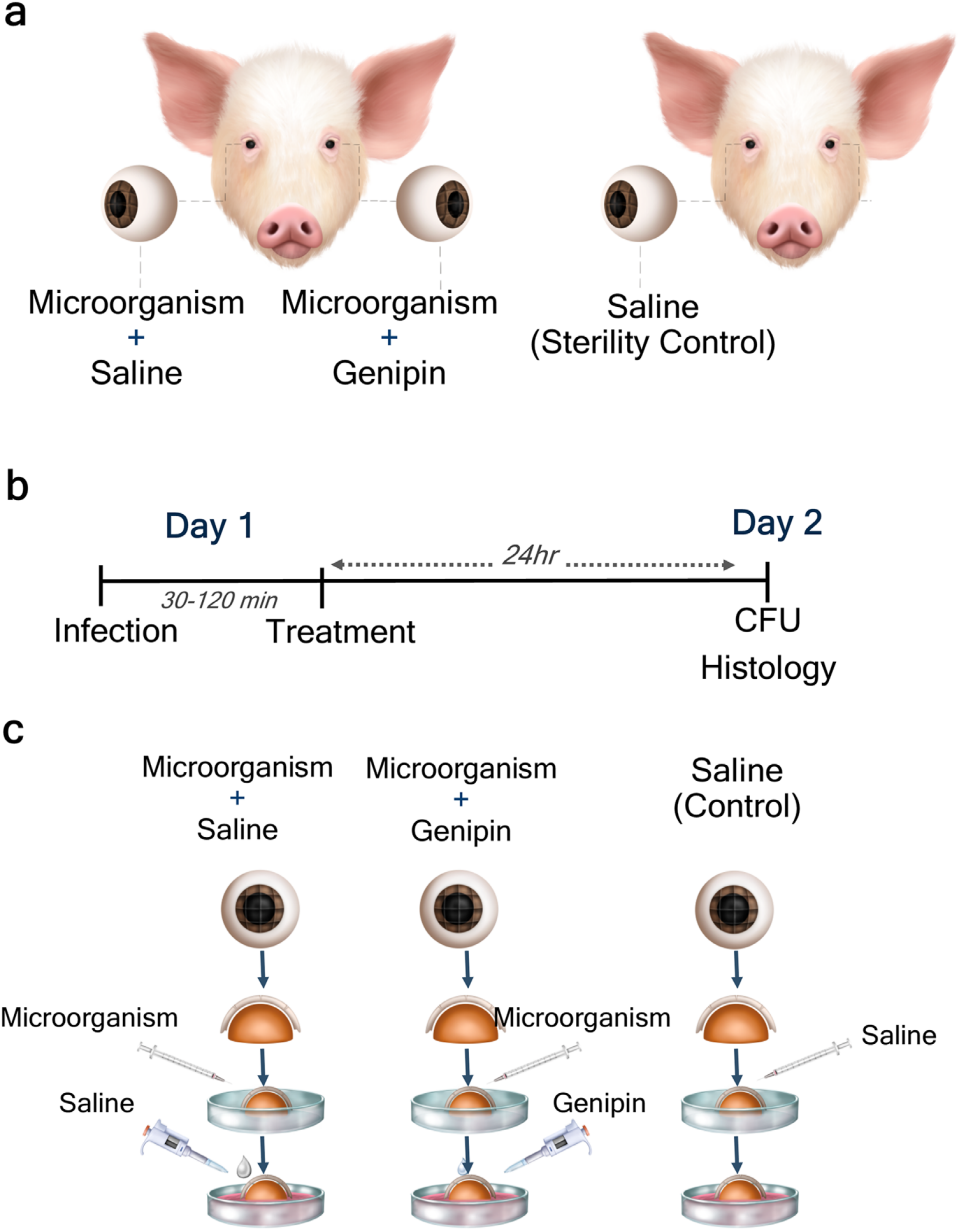
Infection and treatment of the cornea. Schematic representation of the experimental protocol. **(a)** A matched paired study design was implemented and following bacterial inoculation one eye was treated with saline (microorganism + saline) and the contralateral eye with genipin (microorganism + genipin). Wounded but uninfected, untreated corneas from separate animals were used as a sterility control. **(b)** Time schedules of the experiment. On day 1 bacterial infection was established and corneas were treated with saline or genipin for 24 hours. On the following day, CFU analyses (CFU/cornea) and histologic examinations were carried out. **(c)** Illustration of the experimental procedures performed on day 1. To infect the corneas, scalpel incisions were made after which microorganisms were added to the corneal stroma using a syringe. A custom-made, sterile acrylic mold was used for maintaining the shape of the cornea. After 30 minutes of bacterial inoculation or 120 minutes of fungal inoculation, corneas were treated topically with drops of saline solution or genipin.

A schematic representation of the matched paired study design and its time course is shown in Figures 1a and 1b. One eye was randomly selected to receive genipin treatment after corneal infection (microorganism + genipin), whereas the contralateral eye received saline solution after inoculation (microorganism + saline). Wounded, uninfected, and untreated corneas that received saline only (sterility control) allowed validation of the methodology for sterility testing of the cornea and corneal culture (Fig. 1a). After decontamination and scalpel wounding of the cornea as described above, corneoscleral buttons were placed epithelial side up on sterile, custom-made, acrylic hemispheres, conserving the original corneal curvature (Fig. 1c). Using a 27-gauge sterile needle (Becton Dickenson, Bogota, Colombia), a 100 µL aliquot containing 1 × 10^5^ CFU of *S. aureus* or *P. aeruginosa* or 1 × 10^3^ CFU of *C. albicans* was injected into the cornea (Fig. 1c). Untreated corneas (sterility control) were exposed to saline solution only (Fig. 1c). The infected corneas were incubated at 37°C for 30 minutes (bacteria) or 120 minutes (fungi) to allow the pathogens to colonize the cornea. Control corneas were wounded, but not infected, and incubated at 37°C overnight.

After microbial inoculation, the infected corneas were treated with either genipin or saline solution. A single-dose treatment of genipin was used in this study. One eye received dropwise a 100 µL aliquot of the MBC/MFC of genipin against the tested microorganism, determined earlier by the broth dilution method. The contralateral eye received dropwise a 100 µL aliquot of sterile saline solution. Thereafter, Dulbecco's modified Eagle medium (bacterial infection; MERCK, Bogota, Colombia) or RPMI 1640 medium (fungal infection; Gibco Thermofisher Scientific, Bogota, Colombia), was placed in the dish to maintain hydration, and corneas were incubated at 37°C for 24 hours. A total of six pairs of corneas for each bacterial strain and a total of five pairs of corneas for *C. albicans* were used in this study, (excluding the corneas used for sterility controls).

### Microorganism Quantification—Analysis of Bacterial and Fungal Growth (CFU/Cornea)

The following day (24 hours after treatment), microorganism quantification and determination of the CFU/cornea was carried out.^88^^,^^89^ In brief, the cornea was homogenized in 1 mL of sterile saline solution after which the homogenate was serially diluted (10-fold dilutions) in saline solution. Thereafter, 100 µL of each dilution was plated in BHI agar plates at 37°C for 24 hours. The next day, colony enumeration was carried out, and CFU/cornea were expressed as base 10 logarithms, as described elsewhere.^88^

### Histologic Analysis

Cornea was fixed in 10% formaldehyde and processed for histologic analysis. Paraffin-embedded tissue sections (∼5 µm thick) were stained by hematoxylin and eosin (H&E), Gram (bacteria), periodic acid-Schiff (fungi) and Grocott-Gomori's methenamine silver (fungi) stains. Histological sections were observed and imaged using an Olympus microscope (Olympus, Tokyo, Japan) and Canon EOS T4i Rebel camera (Canon Inc., Tokyo, Japan).

### In Vivo Toxicity Assay

The Draize test was used to evaluate potential ocular irritation, as described previously.^90^^,^^91^ This test is validated and adopted by the United Nations Globally Harmonized System (UN GHS) and the European Union Regulation on Classification, Labelling and Packaging regulatory agencies for ocular hazard identification and classification.^92^^,^^93^ In particular, “Category 1” is defined as causing irreversible effects on the eye/serious damage to the eye and “Category 2” as causing reversible effects (fully reversible within 21 days) on the eye. UN GHS further subcategorizes Category 2 into two optional categories: “Category 2A” (irritant to eyes) when the eye effects are not fully reversible within 7 days of observation and “Category 2B” (mildly irritant to eyes) when the eye effects fully reverse within seven days of observation.^92^^,^^93^

New Zealand white rabbits (n = 10; five male and five female), with a weight range between 2.5 and 3.5 kg were used to observe ocular irritation and evaluate the safety of genipin application. Before the eye irritation test, all the rabbits were carefully examined to ensure that the eyes were free of defects and irritation. Briefly, a single dose of 0.1 mL of genipin (15 mM) was applied topically to the right cornea and the contralateral eye served as a control. The ocular tissue condition was observed and evaluated at various time points after application, up to 15 days after treatment. The chemosis, lids closed/open, swelling, discharge and redness of the conjunctiva were graded on a scale from 0 to 3; iris (swelling and hyperemia) was graded on a scale from 0 to 2; and cornea (irritation, opacity until ulceration) was graded on a scale from 0 to 4, as previously described.^94^^,^^95^ In accordance with the UN GHS/ European Union Regulation on Classification, Labelling and Packaging classification system, the sum of the score was then used to classify genipin based on the severity of effects and timing of reversibility of effects.^94^^,^^95^

### Statistical Analysis

Microorganism CFU data were analyzed using SigmaPlot Version 12.0 (Systat Software, San Jose, CA, USA, http://www.sigmaplot.co.uk/products/si-gmaplot/produpdates/prod-updates18.php). A Mann-Whitney U test was performed for CFU/cornea determinations and differences between datasets. Data from the eye irritation test were expressed as mean and standard error. The comparisons between Draize score of control and treated eyes were done using the Mann-Whitney U test. Statistical significance was ascertained at *P* ≤ 0.05.

## Results

### Bactericidal Activity of Genipin Against *S. aureus* and *P. aeruginosa*

**Figure 2.**
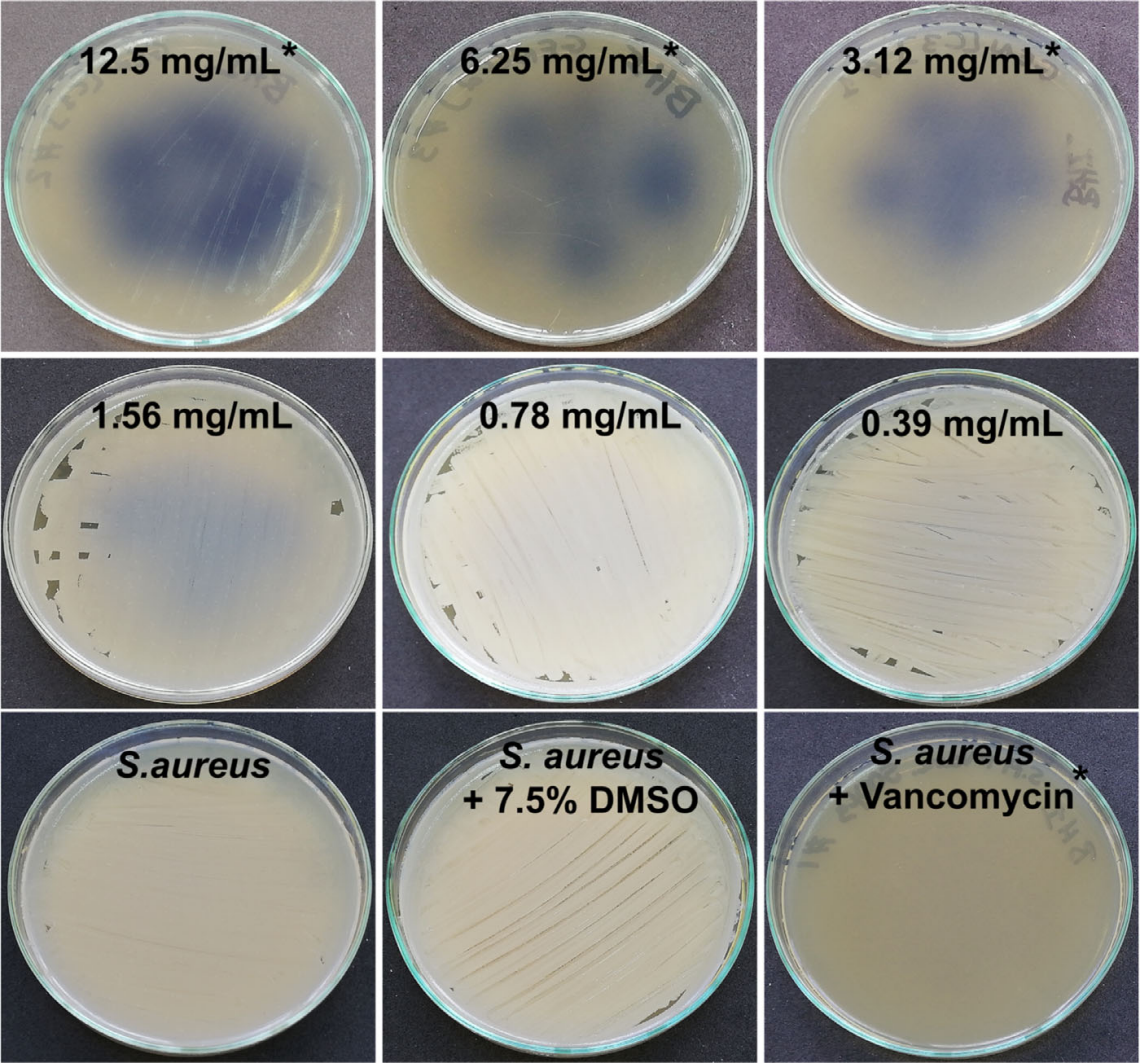
Bactericidal activity of genipin against *S. aureus*. The antibacterial properties of genipin were evaluated using the broth dilution method. Digital photographs of *S. aureus* grown on nutrient agar plates after treatment with different concentrations of genipin for 24 hours. The MBC of genipin against *S. aureus* was 3.12 mg/mL. Untreated bacteria served as a negative control. As a positive control, bacteria were treated with vancomycin. * Denotes no bacterial growth.

**Figure 3.**
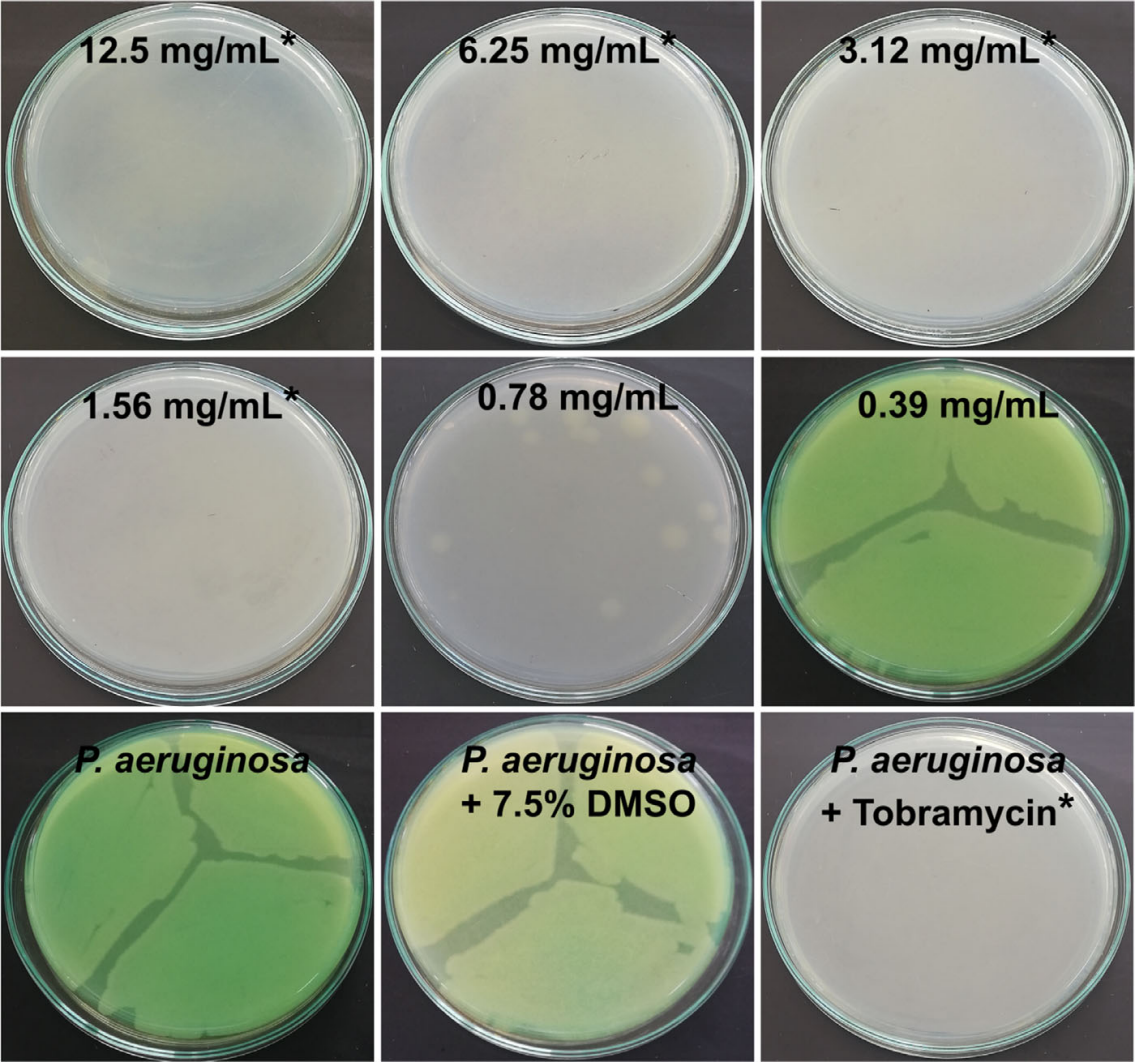
Bactericidal activity of genipin against *P. aeruginosa*. The antibacterial properties of genipin were evaluated using the broth dilution method. Digital photographs of *P. aeruginosa* grown on agar plates after treatment with various concentrations of genipin for 24 hours. The MBC of genipin against *P. aeruginosa* was 1.56 mg/mL. Untreated bacteria served as a negative control. As a positive control, bacteria were treated with tobramycin. * Denotes no bacterial growth.

Along with its effective crosslinking properties, genipin is known to be active against certain Gram-positive and Gram-negative bacteria.^82^^,^^96^ However, its antimicrobial activity against *S. aureus* and *P. aeruginosa*, two of the most frequent causative pathogens of bacterial keratitis,^10^^–^^13^ remains unknown. We hypothesized that these species would likewise be susceptible to genipin and hence investigated the antimicrobial potency of genipin against *S. aureus* and *P. aeruginosa* using the broth dilution method. This demonstrated a potent bactericidal activity against both *S. aureus* and *P. aeruginosa* (Figs. 2 and 3)*.* In the case of *S. aureus*, the MBC value of genipin, confirmed by absence of bacterial growth, was 3.12 mg/mL after 24 hours of incubation (Fig. 2). *P. aeruginosa* was more sensitive to genipin and its MBC value was 1.56 mg/mL after 24 hours of incubation (Fig. 3). Time-kill studies confirmed that the solvent did not affect bacterial growth confirming the antimicrobial activity of genipin against the tested strains (Supplementary Fig. S1, Supplementary Fig. S2). These findings suggest that genipin, although at much higher concentrations than normal, standard antibiotics, has bactericidal effects against common, clinically important Gram-positive and Gram-negative bacteria.

### Genipin Effectively Inhibits the Growth of *S. aureus* in Infected Ex Vivo Porcine Cornea

**Figure 4.**
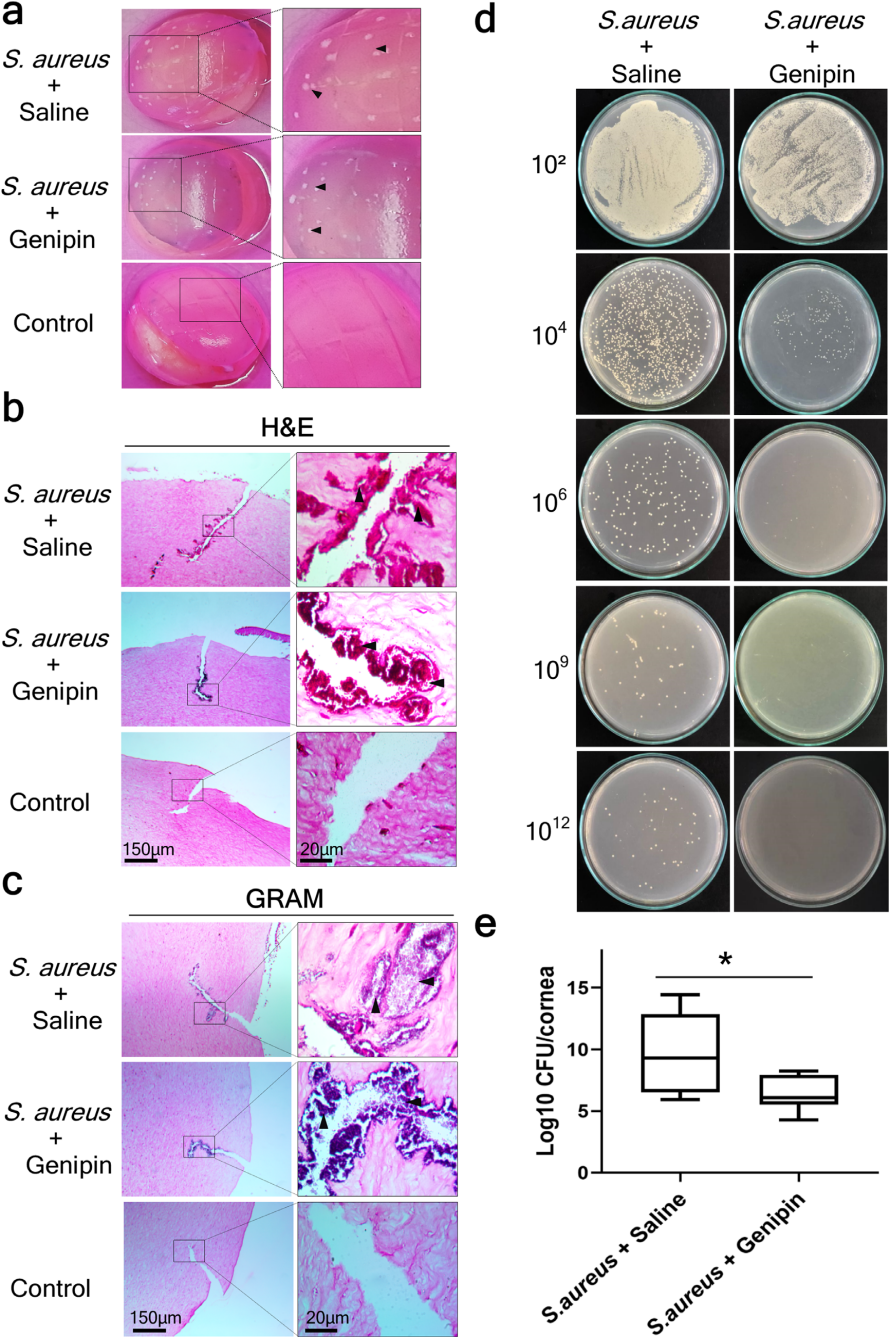
Genipin treatment reduces *S. aureus* growth in the infected ex vivo porcine corneas. **(a)** Macroscopic images of ex vivo corneas infected with *S. aureus* and treated with either saline or genipin for 24 hours demonstrated visible turbidity and bacterial invasion (*arrowheads*). Control corneas were wounded but no bacteria were added and these did not show any macroscopic changes; rather, a clear cornea was maintained. **(b)** Histologic examination of ex vivo porcine corneas with H&E staining showed bacterial corneal stromal colonization (*arrowheads*) exclusively by coccus in both experimental groups (*S. aureus* + saline and *S. aureus* + genipin) and no infection in the sterility control. Gram staining confirmed establishment of infection by Gram-positive bacteria (*arrowheads*) in corneal wounds and corneal stroma in *S. aureus* + saline and *S. aureus* + genipin experimental groups. **(c)** *S. aureus*-infected corneas were homogenized and the suspension was serially diluted and plated onto nutrient agar plates for 24 hours at 37°C. Photographs of CFU in agar plates illustrated notable differences in the bacterial growth between the two groups (*S. aureus* + saline vs *S. aureus* + genipin). **(d)** The number of *S. aureus* colonies, expressed as log was plotted for each treatment group (n = 6 for each experimental group). The *bars* in the box plot represent the minimum and maximum values, whereas the top, middle, and bottom *horizontal lines* depict the upper quartile, median, and lower quartile, respectively. * Statistically significant (*P* < 0.05). All images are representative of six samples per experimental group.

Encouraged by these findings, further investigation was performed to assess the efficacy of genipin against *S. aureus* in an ex vivo porcine model of *S. aureus* keratitis. The infected corneas were treated with genipin at a concentration of 3.12 mg/mL, as determined by the broth dilution experiment, or with saline solution. In vivo, *S. aureus* corneal infections are characterized by epithelial ulceration.^97^ In areas where the microorganisms replicate, microcolonies form and secrete deleterious toxins, killing the epithelial cells and exposing the underlying stroma.^97^ Porcine corneas examined here 24 hours after infection showed a visible haze. Staphylococcal microcolonies had formed in the corneal stroma, but at a reduced number in the genipin-treated corneas relative to the saline-treated group (Fig. 4a). Untreated, sterility control corneas were characterized by a normally clear and transparent cornea (Fig. 4a). To evaluate the distribution of bacteria within the corneal stroma, histologic analysis with H&E and Gram staining was performed 24 hours after treatment. This indicated bacterial penetration of *S. aureus* into infected corneas, with most of the microorganisms located within the incisions (Figs. 4b and 4c). No bacterial colonies were observed in the control corneas (Figs. 4b and 4c). Viable CFU/cornea were counted after 24 hours of incubation as shown in Figure 4d. Comparison of the bacterial CFUs recovered from *S. aureus*–infected corneas revealed that the corneas treated with genipin produced a significantly decreased number of CFUs per cornea (average, log 9.67 ± 1.33 CFU/cornea, n = 6) opposed to the saline-treated corneas (Average, log 6.42 ± 0.59 CFU/cornea, n = 6, *P* = 0.046; Table 1, Fig. 4e).

**Table 1.** Average and Standard Error of the Log_10_ CFU/Cornea of Experimental Groups

	Viable Microbial
	Count (log_10_ CFU
Microorganism	/cornea ± SE)
*S. aureus*	
+ Saline	9.67 ± 1.33
+ Genipin	6.42 ± 0.59^*^
*P. aeruginosa*	
+ Saline	9.37 ± 0.33
+ Genipin	6.72 ± 0.41^*^
*C. albicans*	
+ Saline	3.66 ± 0.25
+ Genipin	2.62 ± 0.50

* Statistically significant, *P* < 0.05.

### Effectiveness of Genipin Against *P. aeruginosa* in the Infected Ex Vivo Porcine Corneal Model

**Figure 5.**
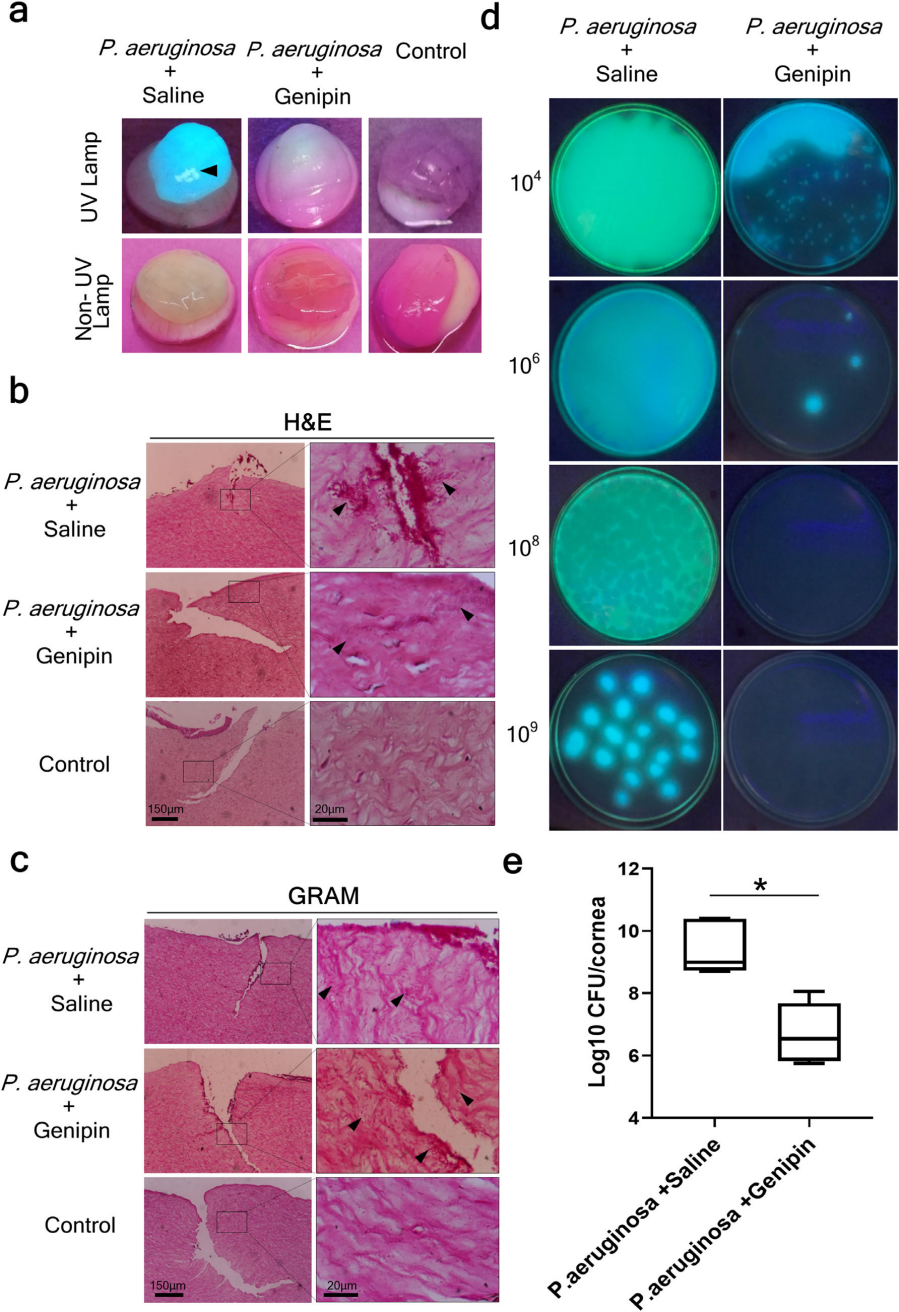
Genipin treatment efficiently reduces *P. aeruginosa* growth in the infected ex vivo porcine corneal model. **(a)** Macroscopic images of ex vivo corneas infected with *P. aeruginosa* and treated with either saline solution or genipin for 24 hours demonstrated visible corneal edema. Infected corneas treated with saline showed bacterial colonization in the corneal surface (*arrowhead*). Fluorescence was observed under UV light, indicating *P. aeruginosa* corneal infection. Control corneas were clear. **(b)** H&E and Gram histologic analysis showed widespread *P. aeruginosa* colonization (rods, *arrowheads*) within the corneal stroma in the *P. aeruginosa* + saline and *P. aeruginosa* + genipin groups. No bacterial infiltration was observed in the control corneas. **(c)** *P. aeruginosa*-infected corneas were homogenized and the suspension was serially diluted and plated onto nutrient agar plates for 24 hours at 37°C. Photographs of CFU in agar plates demonstrated distinct differences in the bacterial growth between *P. aeruginosa* + saline *vs P. aeruginosa* + genipin treated corneas. **(d)** The number of *P. aeruginosa* colonies, expressed as log was plotted for each treatment group (n = 6 for each experimental group). The *bars* in the box plot represent the minimum and maximum values, whereas the top, middle, and bottom *horizontal lines* depict the upper quartile, median, and lower quartile, respectively. * Statistically significant (*P* < 0.05). All images are representative of six samples per experimental group.

As genipin was also shown to inhibit *P. aeruginosa* growth using the broth dilution method, we next investigated its bacteriostatic effect in ex vivo *P. aeruginosa–*infected porcine corneas. Infected corneas were treated with genipin at a concentration of 1.56 mg/mL, the MBC determined earlier by the broth dilution experiment, or with saline solution. Infected corneas exhibited characteristics of *Pseudomonas* keratitis including diffuse tissue surrounding corneal edema and a greenish-yellow discharge (Fig. 5a). Untreated, control corneas were clear and transparent (Fig. 5a). The virulence of *P. aeruginosa* and its ability to rapidly invade the cornea contributes to a fast, progressive and difficult-to-treat keratitis that can lead to vision impairment, sometimes within 72 hours.^17^^,^^98^ Correspondingly, histologic analysis confirmed the rapid penetration of *P. aeruginosa* into the corneal stroma, 24 hours after inoculation, with bacteria being visible within the superficial corneal stroma, as well as within the deeper stroma, located along the collagen lamellae (Figs. 5b and 5c). No bacterial colonies were observed in the control corneas, confirming tissue sterility (Figs. 5b and 5c). Figure 4d photographically demonstrates the results of the CFU analysis. Treatment with genipin successfully produced a significant reduction in the number of CFUs per cornea; averaged log 6.72 ± 0.41 CFU/cornea (n = 6) compared to the saline-treated group, which averaged log 9.37 ± 0.33 CFU/cornea (n = 6, *P* = 0.003, Table 1, Fig. 5e).

### Antifungal Activity of Genipin Against *C. albicans—*Fungal Growth Inhibition in Infected Ex Vivo Porcine Corneas

**Figure 6.**
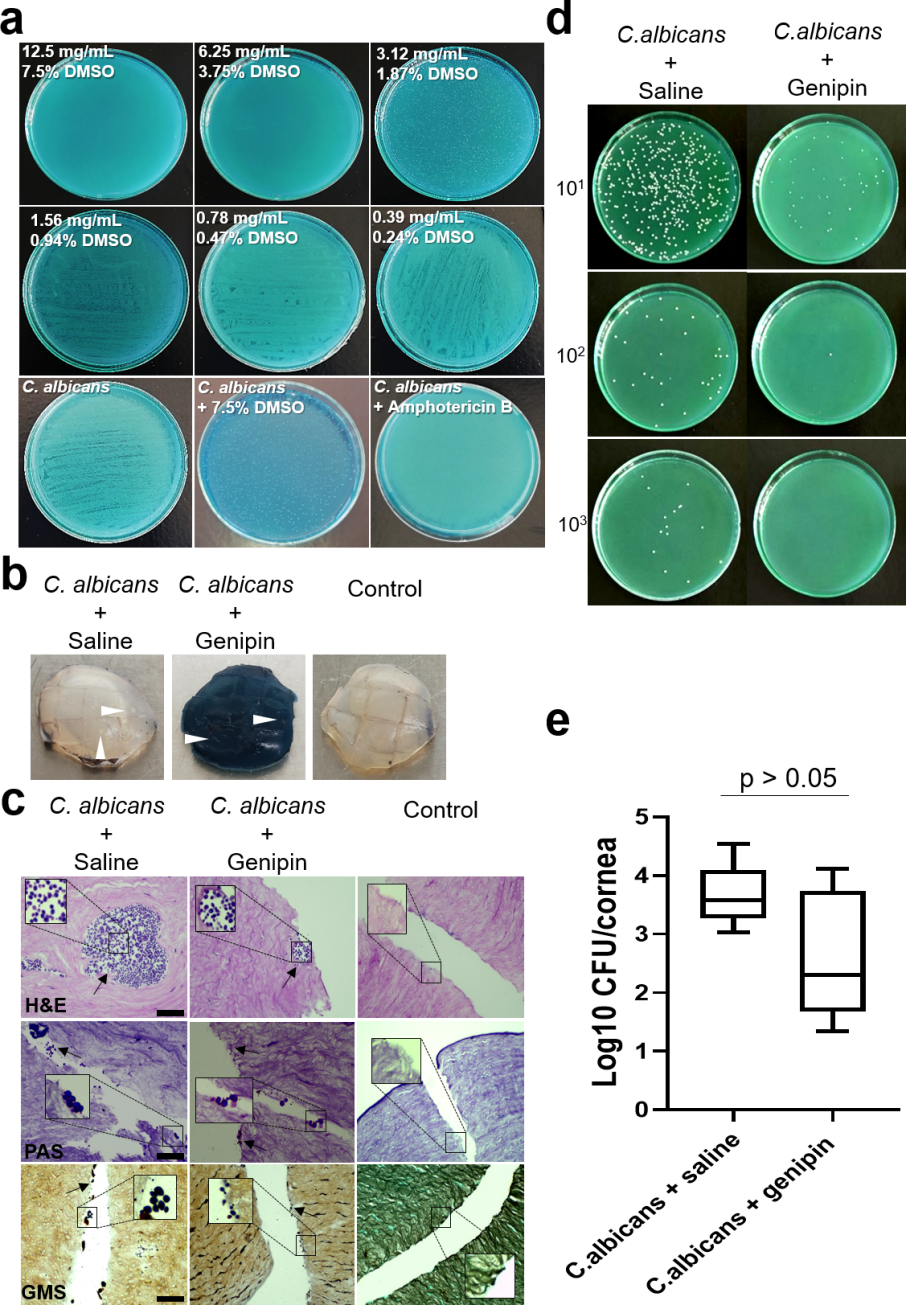
Genipin treatment efficiently reduces *C. albicans* growth in the infected ex vivo porcine corneal model. **(a)** Digital photographs of *C. albicans* grown on nutrient agar plates after treatment with different concentrations of genipin for 24 hours. The MFC of genipin against *C. albicans* was 6.25 mg/mL. **(b)** Macroscopic view of ex vivo corneas infected with *C. albicans* and treated with either saline solution or genipin for 24 hours demonstrated fungal white colonies at the corneal surface. Control corneas were clear and transparent. **(c)** H&E, periodic acid-Schiff, and Grocott-Gomori's methenamine silver histology of infected corneas showed distribution of *C. albicans* within the scalpel wound and into the corneal stroma. No bacterial infiltration was observed in the control corneas. *Scale bar*: 50 µm. **(d)** *C. albicans*-infected corneas were homogenized, and the suspension was serially diluted and plated onto nutrient agar plates for 24 hours at 37°C. Photographs of CFU in agar plates demonstrated distinct differences in the bacterial growth between *C. albicans* + saline vs *C. albicans* + genipin treated corneas. **(e)** The number of *C. albicans* colonies, expressed as log was plotted for each treatment group (n = 5 for each experimental group). The *bars* in the box plot represent the minimum and maximum values, whereas the top, middle, and bottom *horizontal lines* depict the upper quartile, median, and lower quartile, respectively. All images are representative of five samples per experimental group.

Genipin demonstrated antifungal activity against *C. albicans* and specifically was shown to exert fungicidal effect. The MFC value of genipin was 6.25 mg/mL, after 24 hours incubation (Fig. 6a); higher than the one observed for *S. aureus* and *P. aeruginosa*. Time-kill studies validated the nontoxic effect of the solvent against *C. albicans*, (Supplementary Fig. S3). To verify genipin's antifungal activity, its effectiveness was additionally investigated in an ex vivo porcine model of *C. albicans* keratitis. A macroscopic view of the infected corneas demonstrated white yeast colonies on the corneal surface (Fig. 6b, arrowheads). Sterility control corneas were clear and transparent. Histologic examination of *C. albicans–*infected corneas demonstrated round yeast within the scalpel wounds, and into the stroma, particularly in the saline-treated corneas (Fig. 6c). A higher number of *C. albicans* CFU/cornea were recovered in the saline-treated corneas (Average, log 3.66 ± 0.25 CFU/cornea, n = 5), compared to genipin-treated corneas (average, log 2.62 ± 0.50 CFU/cornea, n = 5; *P* = 0.072; Table 1, Figs. 6d, 6e).

### In Vivo Toxicity Assay—Mild Ocular Irritation

**Figure 7.**
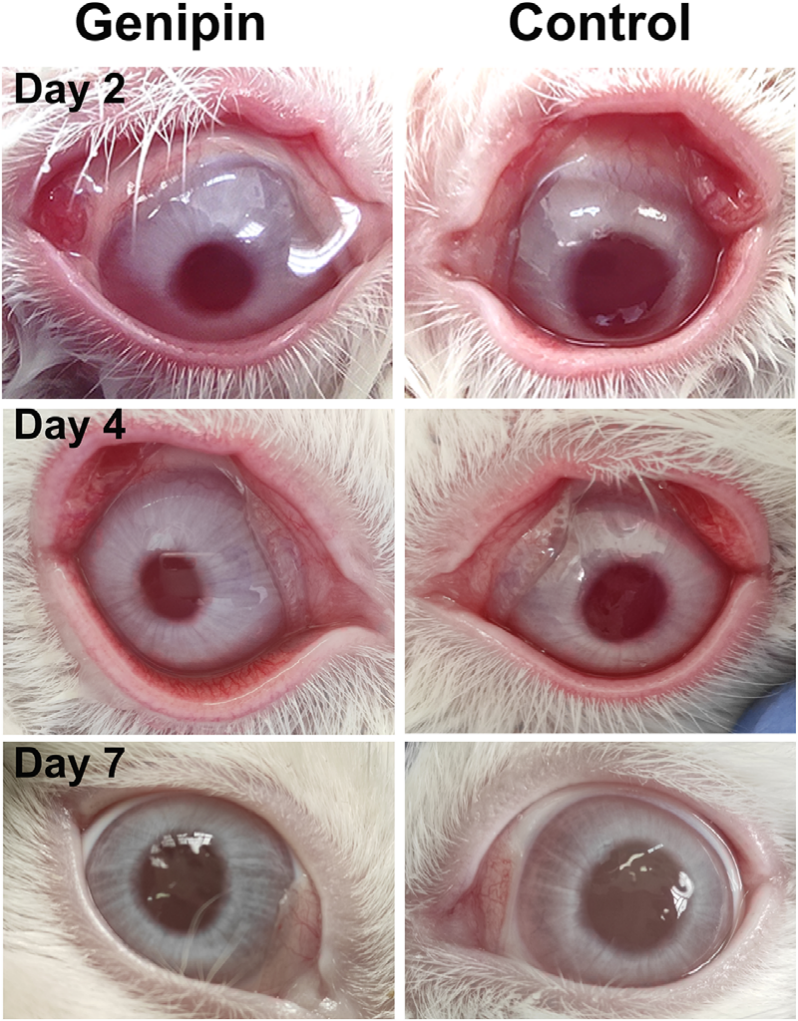
Representative photos of the ocular irritation test. The tested eye was treated with genipin, and the contralateral eye was used as control.

Assessment of the ocular irritation caused by genipin according to the in vivo toxicity assay in rabbits showed a small increase in the conjunctival redness in the first three days (Table 2, Fig. 7). This was followed by a reduction in the score and from days 7 to 15 after application, none of the eyes presented any sequelae (Table 2, Fig. 7). There was no significant difference in the mean score between genipin-treated and control groups five days after application (Table 2, *P* > 0.05). According to these findings, genipin can be considered as a Category 2 irritant and more specifically, Category 2B (mildly irritant to eyes) because the eye effects were fully reversible within seven days of observation.

**Table 2.** Draize In Vivo Toxicity Assay—Eye Test Score

	Genipin	Control	
Day (After Treatment)	Average ± SE	Average ± SE	*P* Value^*^
2	2.3 ± 0.42	1 ± 0.16	0.013
3	1.9 ± 0.29	0.3 ± 0.16	0.001
5	0.3 ± 0.32	0	0.728
7	0	0	0

SE, standard error.

The test scores were obtained by evaluating corneal opacity, irritation, reactivity of iris, conjunctival edema, redness, and ocular discharge.

* *P* < 0.05 statistically significant.

## Discussion

Infectious keratitis is a devastating ocular infection characterized by epithelial defects, underlying stromal inflammation, tissue destruction and corneal melting.^2^^,^^4^^–^^6^^,^^13^ It is a leading cause of serious, irreversible ocular damage and blindness worldwide.^4^^–^^6^ In spite of considerable advances in clinical diagnosis and laboratory investigations, management of infectious keratitis remains a formidable challenge.^19^ In addition, the dramatic global rise of multidrug-resistant bacteria and risk of corneal melting are of major concern and further complicate appropriate treatment and management.^99^^,^^100^ In its “Vision 2020” global initiative, the World Health Organization emphasizes the need for novel, innovative therapeutic approaches, beyond antibiotics, for the management of corneal infectious keratitis, particularly for the treatment of severe and unresponsive cases.^101^ In an effort to meet the urgent need for new antimicrobial drug discovery and development, in recent years a lot of attention has been paid to natural products and medicinal plants due to their low toxicity, pharmacological effects and health benefits.^102^^,^^103^ Among natural product-derived compounds, genipin has received particular attention in ophthalmology because of its effective crosslinking activities.^59^^–^^69^ In this study, the in vitro antimicrobial effects of genipin were evaluated against clinical isolates of *S. aureus, P. aeruginosa and C. albicans* using an ex vivo model of corneal infectious keratitis. Of particular importance, these data support the promising potential of genipin as a novel antimicrobial tool, which could be used for the management of advanced, severe and resistant cases of infectious keratitis.

In evaluating its antibacterial effects, *S. aureus* and *P. aeruginosa* were challenged against different concentrations of genipin and its influence on bacterial cell viability and growth was evaluated. Our findings indicate that genipin exerts bactericidal activity against both strains. Of interest, *P. aeruginosa* was shown to be more sensitive to genipin treatment. This is particularly important as Gram-negative bacteria, owing to the structure of their outer membrane, are intrinsically more resistant to antibiotics and classified as a more serious threat to health and the economy.^104^ Aside from their structural differences, a variation in sensitivity between the tested microorganisms might be also attributed to discrepancies in their chemical properties and diversities in metabolism and metabolic pathways.^105^^–^^107^ Previous studies have shown antifungal activity of genipin against two plant pathogenic fungi, *Fusarium oxyspor*um and *Corynespora cassiicola*.^108^ In this study, genipin treatment reduced the concentration of *C. albicans* by one order of magnitude representing a 90% reduction in fungal population; however, this is not considered significant in microbiology as it does not fulfil the recommended 3 log reduction principle.^109^

To better evaluate the antimicrobial efficacy of genipin against these microorganisms, microbial burden was investigated in an ex vivo porcine model of infectious keratitis. Our data demonstrated that a one-time treatment of genipin rapidly and significantly reduced both the bacterial CFU and fungal CFU in the infected corneas in ex vivo models of *S. aureus, P. aeruginosa,* and *C. albicans* corneal keratitis. These results further support the antibacterial properties of genipin and are in agreement with previous studies indicating that genipin possesses bactericidal activity against Gram-positive and Gram-negative bacteria.^96^ Importantly, genipin has recently been shown to suppress *Helicobacter pylori* infection by interfering with the growth and virulence of the pathogen, as well as by attenuating the gastric inflammation caused by the infection.^96^ Having bactericidal action has immense clinical importance, first, because in theory, rapid bacterial elimination results in early resolution of the disease and a better clinical outcome and, secondly, because eliminating bacteria diminishes the emergence of bacterial resistance and spread of disease. Moreover, although fungal resistance is not as uncontrolled as bacterial resistance, the economic aspects associated with fungal infections are incredibly high.^110^ Therefore genipin is presented as a promising antibacterial and antifungal agent that might be useful for the treatment of patients suffering from corneal infection, although further in vivo experiments need to be performed to assess the efficacy and safety.

Although the exact mechanisms of action of genipin are yet to be elucidated, it is likely that its antimicrobial activity is multifaceted and might be attributed to its crosslinking properties.^55^^,^^59^^,^^60^^–^^64^ This is particularly advantageous because, unlike traditional treatment approaches that focus on direct microbial killing, genipin corneal crosslinking offers a dual modality to treat and manage infectious keratitis. In addition to direct bacterial killing, as a natural crosslinker, one conceivable microbial killing mechanism is crosslinking of various proteins present at the surface of the pathogen, such as peptidoglycan, lipoteichoic acid and lipopolysaccharide (LPS).^111^^–^^113^ Thereby, electrostatic interactions with chemical moieties of the bacterial cell wall or membrane may interfere with cellular processes such as division/growth and essentially eradicate bacteria via a membrane damage action mechanism. Another possible killing mechanism of action is that after membrane disruption and alteration in membrane permeability, genipin might additionally interact with intracellular enzymes and nucleic acids and in turn disrupt key metabolic functions, rendering bacterial survival and replication impossible.^111^^–^^113^ In a similar manner, the antifungal activity of genipin might be attributed to fungal cell membrane interference, modifying structural-functional properties and ultimately affecting spore germination, proliferation, and cellular respiration.^108^

Microbial virulence and pathogenicity are generally determined by the ability of the microorganism to invade and colonize the tissue, resist the host defense response, and induce tissue damage.^13^ The adherence of Gram-positive bacteria, including *S. aureus*, relies on their attachment to host extracellular matrix molecules, such as collagen and fibronectin.^114^^,^^115^ On the other hand, *P. aeruginosa* binds to sialylated glycoproteins,^116^^,^^117^ and recent studies have shown that the organism uses the specific arrangement of aligned collagen lamellae to facilitate quick migration through the corneal stroma.^118^ With regard to adherence of fungal pathogens in host cells, potential fungal binding sites include laminin, fibronectin, and collagen.^13^ Hence, besides direct microbial killing through crosslinking and interference with vital cellular functions, by virtue of corneal collagen crosslinking, genipin might also target pathogen virulence by restraining microbial adhesion and spread on the host cell surface.

The ocular response against infectious keratitis induces the secretion of several inflammatory cytokines, such as IL-1a, IL-1β, IL-6, IL-8, tumor necrosis factor α, and the infiltration of immune cells at the site of infection.^119^^,^^120^ Intense inflammation in response to bacterial keratitis, hence, needs to be controlled. One of the most important virulence factors of *P. aeruginosa* is LPS because it mediates the pathogen's adherence and survival within the cornea and stimulates host inflammatory responses, causing robust cytotoxic damage to the cornea.^120^^–^^122^ The important role of genipin in attenuating LPS-induced inflammation has been noted in several tissues,^72^^,^^123^^–^^126^ along with its anti-inflammatory and antioxidant properties.^72^^–^^74^ This notion is further supported by recent studies by Chang and colleagues,^96^ who demonstrated that genipin inhibits the secretion of proinflammatory cytokines IFN-γ and IL-8, as well as LPS-induced oxidative stress in *Helicobacter pylori* infection. However, it remains to be determined whether genipin exerts similar anti-inflammatory effects in corneal infectious keratitis.

Previous studies have shown that genipin-crosslinked corneas are markedly more resistant to enzymatic digestion.^61^^–^^64^ Bullous keratopathy, a type of corneal edema, represents a predisposing factor for microbial keratitis.^127^ Intriguingly, the clinical therapeutic effects of genipin crosslinking in decreasing corneal edema and improving corneal epithelial erosion have been recently demonstrated in vivo, in a rabbit bullous keratopathy model, suggesting that treatment with genipin is rendering the cornea less susceptible to microbial proteolytic enzymes and tissue destruction.^128^ The in vivo toxicity assay displayed minor ocular irritation with the eye effects being fully reversible within seven days of observation, further supporting that genipin can be classified as mildly irritant to eyes.

In conclusion, the current study investigated the antimicrobial potential of genipin against *S. aureus, P. aeruginosa* and *C. albicans* in an ex vivo corneal model of infectious keratitis. Our data demonstrate that genipin exhibits notable in vitro antibacterial and antifungal activity against *S. aureus, P. aeruginosa* and *C. albicans* and raises the possibility of using genipin corneal crosslinking as a novel, innovative therapeutic approach for the treatment and management of infectious keratitis, especially in advanced cases that are nonresponsive to conventional therapy. In vivo studies are necessary to evaluate the action and penetration profile of genipin. One emphasis of future research will be in vivo pharmacokinetic/pharmacodynamic integration and modeling studies of genipin to optimize dosage schedule delivery and application. The potential application of genipin as eye drops is massively advantageous over alternative therapeutic strategies like photodynamic therapy because it does not need the use of light, being applicable to affected patients with very thin corneas that are not eligible for this therapeutic regiment, it is cheaper and can be easily used in developing countries where the major risk for infectious keratitis is trauma due to agricultural work and not having easy access to hospitals. Although various potential mechanisms of action have been put forward to explain the antimicrobial effects of genipin, further research is required to fully elucidate the exact underlying mechanisms as to how bacterial cell death is accomplished. Clearly, a full understanding of both the wider antimicrobial activity of genipin and also its promising anti-inflammatory activity will help considerably in evaluating the importance and potential advantages of genipin therapy in clinical practice. This would have enormous clinical and scientific implications because it could lead to the establishment of a novel therapeutic algorithm for infectious keratitis in an era where antimicrobial resistance is a global concern.

## Supplementary Material

Supplement 1

Supplement 2

Supplement 3
