# Supplementary figures and images for: Genipin in an Ex Vivo Corneal Model of Bacterial and Fungal Keratitis
Source: Transl Vis Sci Technol. 2021 Aug 26;10(9):31. doi: 10.1167/tvst.10.9.31 (PMC8399543; doi:10.1167/tvst.10.9.31)

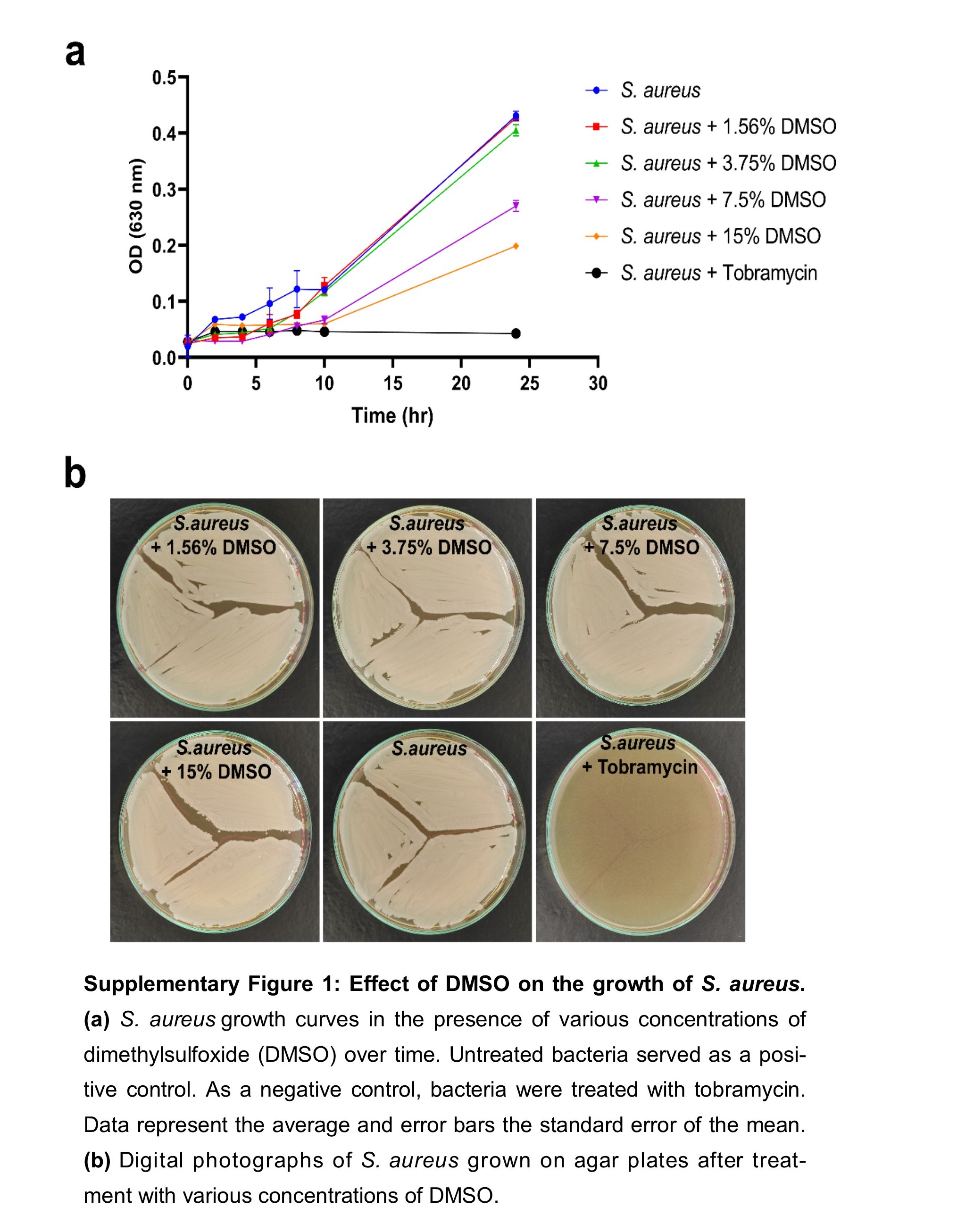

Supplement: Supplement 1 [file tvst-10-9-31_s001.jpg]

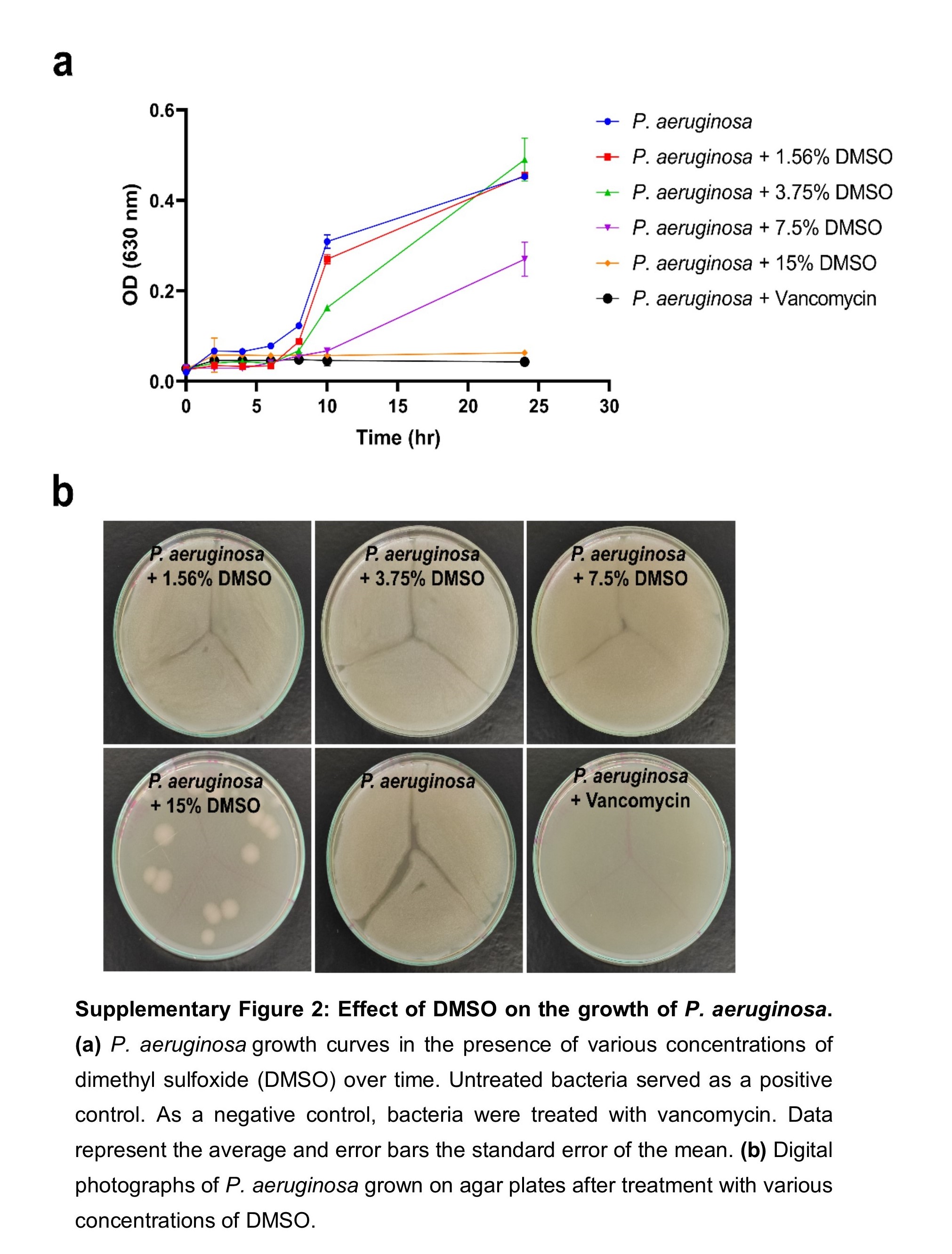

Supplement: Supplement 2 [file tvst-10-9-31_s002.jpg]

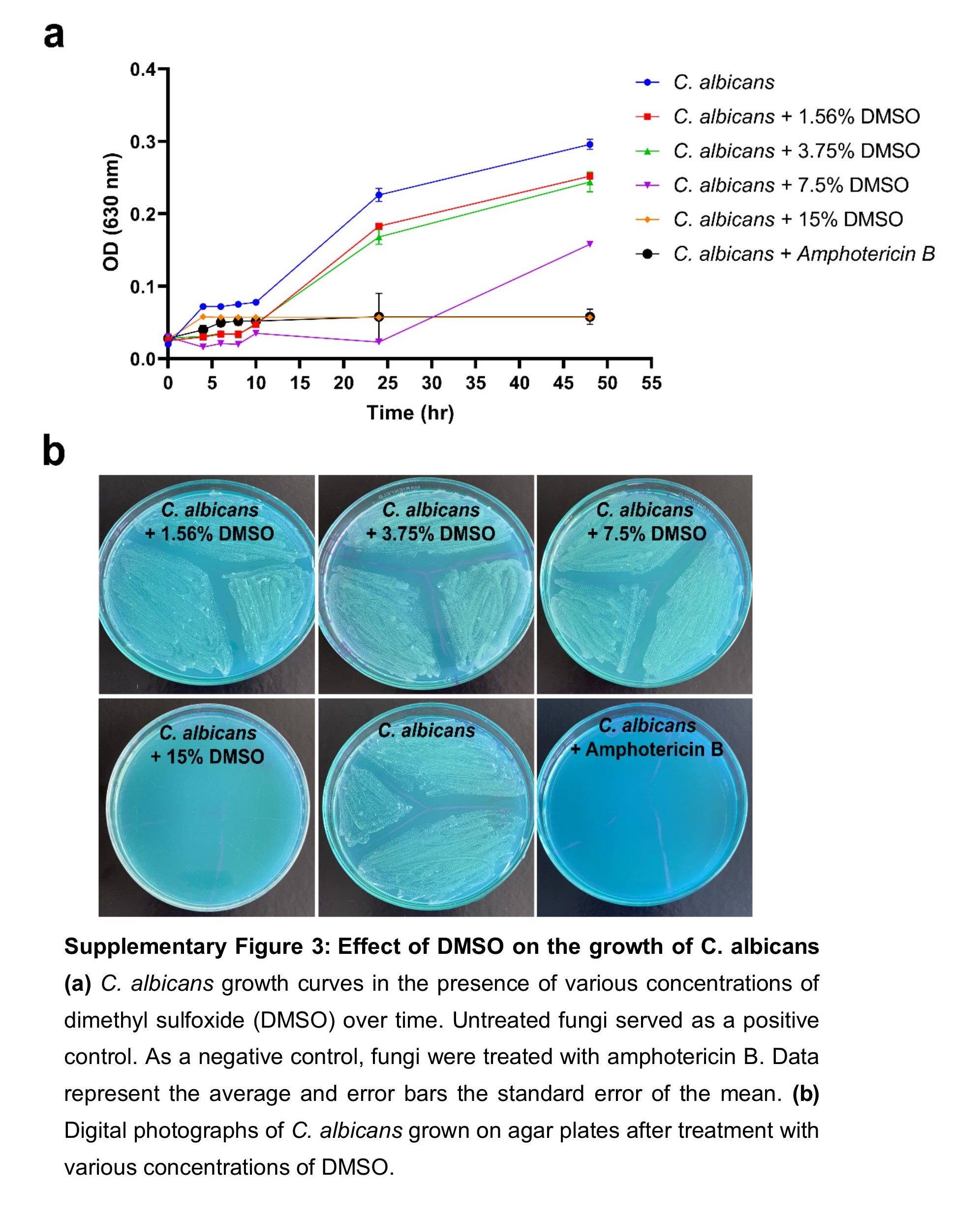

Supplement: Supplement 3 [file tvst-10-9-31_s003.jpg]
